# Incidence and burden of long COVID in Africa: a systematic review and meta-analysis

**DOI:** 10.1038/s41598-023-48258-3

**Published:** 2023-12-06

**Authors:** Luisa Frallonardo, Francesco Vladimiro Segala, Kajal D. Chhaganlal, Mohmaoud Yelshazly, Roberta Novara, Sergio Cotugno, Giacomo Guido, Roberta Papagni, Agnese Colpani, Andrea De Vito, Mario Barbagallo, Giordano Madeddu, Sergio Babudieri, Peter Lochoro, Jerry Ictho, Giovanni Putoto, Nicola Veronese, Annalisa Saracino, Francesco Di Gennaro

**Affiliations:** 1 Clinic of Infectious Diseases, Department of Precision and Regenerative Medicine and Ionian Area (DiMePRe-J), University of Bari “Aldo Moro”, 70124 Bari, Italy; 2 Department of Research, Faculty of Health Sciences, Universidade Catolica de Mocambique, Beira, Mozambique; 3 Unit of Infectious and Tropical Diseases, Department of Medicine, Surgery and Pharmacy, University of Sassari, Sassari, Italy; 4 Doctors with Africa, CUAMM, Kampala, Uganda; 5 Geriatrics Section, Department of Internal Medicine, University of Palermo, Palermo, Italy; 6 Operational Research Unit, Doctors with Africa CUAMM, Padua, Italy

**Keywords:** Quality of life, Diseases

## Abstract

Long COVID, also known as “post-acute sequelae of COVID-19,” affects at least 65 million individuals worldwide with a wide spectrum of symptoms that may last weeks, months, or permanently. Its epidemiology and burden in Africa are unclear. This meta-analysis examines long-term COVID-19 effects in the WHO African Region. A systematic search in several databases was carried out up to 12 February 2023 including observational studies from African countries reporting the cumulative incidence of long COVID signs and symptoms. Only studies conducted in African countries were included. Several sensitivity and meta-regression analyses were performed. Among 1547 papers initially screened, 25 were included, consisting of 29,213 participants. The incidence of any long COVID symptomatology was 48.6% (95% CI 37.4–59.8) as psychiatric conditions were the most frequent, particularly post-traumatic stress disorder reaching a cumulative incidence of 25% (95% CI 21.1–30.4). Higher age (*p* = 0.027) and hospitalization (*p* = 0.05) were associated with a higher frequency of long COVID. Long COVID poses a significant burden in Africa, particularly concerning psychiatric conditions. The study recommends identifying at-risk people and defining treatment strategies and recommendations for African long-COVID patients. High-quality studies addressing this condition in African setting are urgently needed.

## Introduction

In October 2021, the World Health Organization (WHO) provided a consensus definition of long COVID as a condition lasting at least two months in individuals diagnosed with confirmed or presumptive acute SARS-CoV2 infection three months before^1^. The global incidence of long COVID is around 10% of affected people, with approximately 65 million cases worldwide^2^. Systematic reviews demonstrated its impact in terms of disability, activity impairment, cognitive function and overall quality of life^3, 4^, with a global pooled prevalence of quality of life impairment ranging from 38 to 63%^5^. However, in low-income countries, the estimates of its incidence vary greatly due to a significant number of hidden infections (i.e., asymptomatic or undisclosed) and difficulties in accessing testing^6^. Up to June 2023, 9.5 million cases of COVID‐19 have been recorded across the 47 countries of the WHO Afro Region, with more than 175.000 deaths^7^ but, despite the administration of 1084.5 million doses among 1137.4 million doses received, fewer than 51.8% of the people are fully vaccinated^8^.

Risk factors for the occurrence of signs and symptoms of long COVID are older age, comorbidities, anti-SARS-CoV2 vaccination status, hospitalization and progression towards severe acute COVID-19^9^. Along with reducing the risk of progression towards severe or critical COVID-19, vaccination against SARS-CoV2 correlates with a lower incidence and severity of post-COVID conditions^9–13^. Nevertheless, even with the increasing evidence available, the understanding of the impact of this condition in low- and middle-income countries (LMICs) remains uncertain, and there is a notable lack of knowledge regarding the epidemiology and burden of post-acute sequelae of COVID-19 in Africa. Consequently, the objective of this systematic review and meta-analysis is to comprehensively examine the occurrence of post-acute sequelae of COVID-19 in the African continent. Additionally, we aim to evaluate the burden of this condition in terms of prevalent symptoms and risk factors, in order to advocate for well-structured initiatives that facilitate appropriate care for affected individuals. By undertaking this investigation, we hope to enhance the understanding and management of post-acute sequelae of COVID-19 in Africa.

## Materials and methods

### Protocol registration

This study was conducted following the recommendations in the Cochrane handbook for systematic literature reviews to conduct the screening and selection of studies^14^. This systematic review and meta-analysis was reported following the Preferred Reporting Items for Systematic Reviews and Meta-Analyses (PRISMA) guidelines, updated version to 2021^15^. The protocol has been registered in Prospero (Number Registration n°CRD42023397445).

## Research question

The research question for this systematic review is: “What is the incidence of long COVID signs and symptoms in Africa?” To guide the identification of adequate keywords to build search strategies to search bibliographic databases, the research question was framed into the PICO(S) (Participants, Intervention, Comparison, Outcome, Study design) format: (P) laboratory confirmed and/or clinically diagnosed COVID-19: long COVID was defined as the presence of signs and/or symptoms cannot be explained by other medical conditions; (I): none; (C) none; (O) incidence of signs and symptoms of long COVID in African countries; (S) observational studies.

### Information sources and search strategies

We searched Medline (via Ovid) and Web of Science from database inception to 08 February 2023. The search for individual studies in these bibliographic databases was supplemented by a manual search of references included in relevant systematic reviews already published regarding this topic.

Considering the main PICOS elements, we built the following search strategy for Medline: “(Africa OR Angola OR Algeria OR Benin OR Botswana OR Burkina Faso OR Burundi OR Cameroon OR Cape Verde OR Chad OR Central African Republic OR Comoros OR Ivory Coast OR Congo OR Egypt OR Eritrea OR Ethiopia OR Gabon OR Gambia OR Ghana OR Djibouti OR Guinea OR Kenya OR Lesotho OR Liberia OR Libya OR Madagascar OR Malawi OR Mali OR Mauritania OR Mauritius OR Morocco OR Mozambique OR Namibia OR Niger OR Nigeria OR Rwanda OR “São Tomé and Príncipe” OR Senegal OR Seychelles OR Sierra Leone OR Somalia OR South Africa OR Sudan OR eSwatini OR Tanzania OR Togo OR Tunisia OR Uganda OR Zambia OR Zimbabwe) AND (“COVID-19” OR “Novel Coronavirus–Infected Pneumonia” OR “2019 novel coronavirus” OR “2019-nCoV” OR “SARS-CoV-2”) AND (“lingering symptoms” OR “persistent symptoms” OR “long-term symptoms” OR “long-term Covid” OR “long-term” OR “long term” OR “long”)”. Then we adapted the search strategy for Web of Science. The management of potentially eligible references was carried out using the Rayyan website (https://www.rayyan.ai/).

### Eligibility criteria

Inclusion criteria comprised the following: (1) observational studies (case–control, cohort, longitudinal studies); (2) studies that investigated the diagnosis of long COVID according to all diagnostic criteria and follow-up time; (3) studies made in Africa from March 2020 to February 2023. Only articles written in English were included. Studies with an unclear follow-up, case series and case reports were excluded.

### Study selection

We followed the recommendations reported in the Cochrane handbook for Systematic reviews to select studies that were finally included in this review^14^. The selection of the articles was performed independently by six authors (ADV, RN, LF, AC, BZ, RP), in couples. Consensus meetings were held with all reviewers to discuss the studies for which divergent selection decisions were made. Two additional senior members (FVS, FDG) of the review team were involved, when necessary. The studies selection process involved, first, a selection based on title and/or abstracts, then a selection of studies retrieved from this first step based on the full-text manuscripts.

### Data collection and data items

We collected the following information: data regarding the identification of the manuscript (e.g., first author name and affiliation, year of publication, journal name, title of the manuscript), data on the characteristics of the population considered (e.g., sample size, mean age, country, gender, etc.), setting (e.g., hospital, intensive care unit, etc.), method of follow-up visit, follow-up in months, type of diagnosis of COVID-19, number of people vaccinated, hospitalized or admitted in intensive care unit, type of variant, and signs and symptoms recorded during the follow-up period. These data were collected using a standard data extraction form in Microsoft Excel. The data extraction was carried out independently by the six authors, in couples, with one author for each couple extracting the data and the other checking, with the senior authors checking the quality of the data extraction.

### Risk of bias evaluation

The Newcastle–Ottawa Scale (NOS) was used to assess the study quality/risk of bias^16^. The NOS assigns a maximum of nine points based on three quality parameters: selection, comparability, and outcome. The evaluation was made by one author and checked by another, independently. The risk of bias was then categorized as high (< 5 points), moderate (6–7), or low (8–9)^17^. The investigators solved any discrepancies by jointly re-assessing an article (NV and FDG).

### Data synthesis and analysis

Signs and symptoms were grouped into anatomical clusters, as proposed in Di Gennaro et al.^18^. The cumulative presence of symptoms and signs and 95% confidence intervals (CIs) were estimated using a meta-analysis, under a random-effect model^19^. Heterogeneity between estimates was assessed using the I^2^ statistic. In case of an I^2^ over 50% a series of meta-regression analyses (taking as moderators if the participants were hospitalized or admitted to ICU, the percentage of females, and the mean age of the sample size) was conducted. Several sensitivity analyses (hospitalization, admitted to ICU, follow-up mode, and sub-continents) were also run. Moderators and strata were chosen based on clinical judgment. Publication bias was assessed by visually inspecting funnel plots and using Egger bias test, with a *p* value < 0.05 indicative of possible publication bias^20^. All analyses were performed using “metaprop”, a command available in STATA 14.0.

## Results

### Literature search

**Figure 1:**
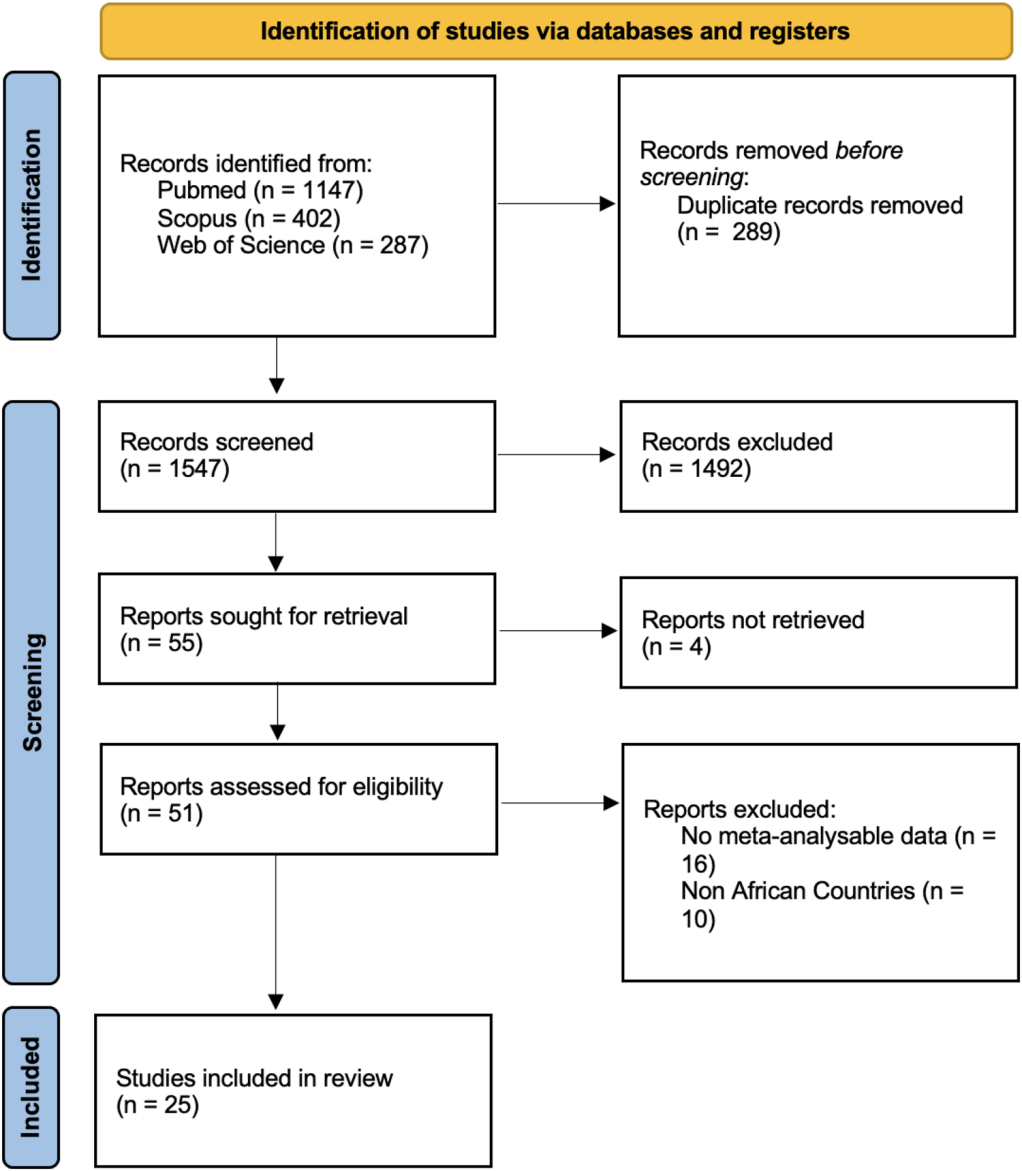
PRISMA 2020 flow diagram for new systematic reviews which included searches of databases, registers and other sources. *Consider, if feasible to do so, reporting the number of records identified from each database or register searched (rather than the total number across all databases/registers). **If automation tools were used, indicate how many records were excluded by a human and how many were excluded by automation tools. *From:* Page MJ, McKenzie JE, Bossuyt PM, Boutron I, Hoffmann TC, Mulrow CD, et al. The PRISMA 2020 statement: an updated guideline for reporting systematic reviews. BMJ 2021;372:n71. https://doi.org/10.1136/bmj.n71. For more information, visit: http://www.prisma-statement.org/

The flow-chart of this systematic review is shown in Fig. 1. Overall, we retrieved 1836 papers and, after excluding duplicates, we screened 1547 works based on title and abstracts. We then evaluated the full text of 55 work, finally including 25 papers.

### Descriptive characteristics

Altogether, the 25 studies included a total of 29,213 African participants. Supplementary Table 1 shows the main descriptive characteristics of the studies included. The studies were mainly made in Egypt (12/25 = 48%), their mean age was 42 years (range 7–73) and the percentage of females was 59.3%. The principal method for diagnosis COVID-19 was PCR (17/25 = 68%). The follow-up method preferred for assessing long COVID was in person visits (n = 9), followed by online survey (n = 8). The median follow-up time was 3 months (range 0.5–12). Vaccination status was mainly unknown as well as the main COVID-19 variant (Supplementary Table 1).

### Risk of bias

The Newcastle–Ottawa Scale (NOS) was used to assess the study quality/risk of bias. The NOS assigns a maximum of 9 points based on three quality parameters: selection, comparability, and outcome. The evaluation was made by one author and checked by another, independently. The risk of bias was then categorized as high (< 5 points), moderate (6–7), or low (8–9)^17^ (Supplementary Table 1).

### Presence of long COVID symptomatology

As shown in Table 1, among the 25 studies that included 29,213 African participants previously affected by COVID-19 the cumulative incidence of long COVID was 48.6%, overall indicating that about half of the patients included had long COVID.

**Table 1:** Cumulative incidence of long COVID signs and symptoms in Africa.

System	Number of cohorts	Total sample size	Cumulative incidence	95% CI
Any	**25**	**29,213**	**48.6**	**37.4–59.8**
Neurological				
Headache	14	10,515	12.7	9.8–15.5
Taste disorder (ageusia or dysgeusia)	11	9128	7.1	5.0–9.1
Smell disorder (anosmia)	12	9926	6.7	5.0–8.4
Cognitive impairment	4	2448	15.0	10.2–19.7
Memory deficits	3	2148	7.7	4.3–11.1
Difficulty concentrating	4	5635	10.7	5.5–16.0
Dizziness	9	8782	6.7	4.2–9.2
Tremors	2	812	1.2	0.4–1.9
Seizures	2	4499	0.2	0.05–0.25
Cramps	1	538	2.6	1.6–4.3
Visual impairment	5	6134	7.4	3.7–11.1
Psychiatric				
PTSD	2	341	25.8	21.1–30.4
Depression	10	18,811	18.2	10.9–25.4
Sleep disorders	13	8333	20.3	15.8–24.8
Anxiety	8	3171	24.4	18.0–30.8
Respiratory				
Cough	9	7836	10.7	7.5–13.8
Dyspnea	12	8057	18.3	12.2–24.4
Nasal congestion	2	3700	1.9	1.4–2.3
Voice change	1	115	5.2	2.4–10.9
Mobility issues				
Mobility impairment	2	3700	1.4	1.0–1.8
Mobility decline	2	3700	0.9	0.6–1.2
Heart				
Palpitations	8	5771	11.0	7.4–14.6
Digestive				
Abdominal pain	9	7210	6.1	4.0–8.1
Diarrhea	8	6908	6.2	4.3–8.2
Vomit	5	2626	1.5	0.6–2.3
Loss of appetite	6	4929	12.7	9.0–16.4
Skin				
Rash	6	6400	2.3	1.2–3.3
Hair loss	3	3872	3.5	1.7–5.3
General				
Weight loss	4	2726	10.4	4.2–16.7
Constitutional				
Myalgia	11	7364	15.5	11.1–19.9
Pain	11	8590	11.1	8–14.1
Fever	7	6974	9.9	6.7–13.0
Fatigue	15	10,577	35.4	25.6–45.2
Arthralgia	10	7285	17.3	12.4–22.2
Sore throat	6	1493	5.7	2.4–9.0
Sweats	2	446	4.6	2.7–6.5
Poor QoL	1	174	25.3	19.4–32.2

Data are reported as cumulative incidence with their 95% confidence intervals.

*PTSD* post-traumatic stress disorder, *QoL* quality of life.

Significant values are in bold.

Psychiatric conditions were the most frequent symptomatology among long COVID signs and symptoms, with post-traumatic stress disorder reaching a cumulative incidence of 25.8% (95% CI 21.1–30.4). Among neurological signs and symptoms, the most frequent was cognitive impairment present in 15% of the participants included. Dyspnea was the most frequent respiratory symptom reported (18.3%) followed by cough (10.7%), while palpitations were more frequent among cardiac symptomatology (Table 1). Of importance, loss of appetite (12.7%) and weight loss (10.4%) were extremely common among gastrointestinal and general signs and symptoms as well as fatigue (35.4%) myalgia (15.5%) (Table 1). Overall, self-reported poor quality of life (25.4%) was extremely frequent.

### Meta-regression and sensitivity analyses

Since the heterogeneity observed of any sign and symptom was 99%, we did run several sensitivity and meta-regression analyses. Table 2 shows the main meta-regression analyses of our investigation. Among the moderators considered, every one-year increase in age was associated with a significantly higher probability of 10% in having any sign or symptom of long COVID. Higher mean age explained 21.4% of the heterogeneity observed. Similarly, an increase of 1% of hospitalized people was associated with a higher presence of 0.3% of any long COVID symptomatology during the follow-up (Table 2). This factor explained the 15.8% of heterogeneity observed. Other factors considered, such as higher percentage of females, duration of follow-up or higher percentage of admissions in intensive care units were not able to explain any heterogeneity.

**Table 2:** Meta-regression analysis of any long COVID signs and symptoms.

Moderator	Beta	SE	*p* value	R2
% of females	0.004	0.004	0.41	0.00
Mean age	**0.10**	**0.04**	**0.027**	**21.4**
Duration of the follow-up	0.03	0.02	0.17	5.0
Percentage of hospitalized	**0.003**	**0.001**	**0.048**	**15.8**
Percentage ICU	0.002	0.004	0.64	0.00

Data are reported as Beta (B) and their standard error and correspondent p-values and adjusted R2. The beta coefficient represents the change in the dependent variable (in this case, the presence of any long COVID signs or symptoms) associated with a one-unit change in the independent variable.

Significant values are in bold.

Table 3 reports the sensitivity analyses for the main outcome of our investigation, i.e., presence of any long COVID symptomatology. The presence of long COVID seems not to be dependent on hospitalization or admission in ICU or follow-up mode (*p* for interaction > 0.05). On the contrary, we observed that long COVID was more frequent in Northern (47.73%) or Southern (48.89%) African countries when compared to Eastern ones (5.06%) (Table 3).

**Table 3:** Sensitivity analyses for long COVID symptomatology in Africa.

Moderator	Strata	Prevalence	95%CI		*p* for interaction
Hospitalization	Yes	56.38	31.07	81.69	0.57
	No	34.02	0.00	89.33	
	Mixed	47.73	36.77	58.69	
Admitted to ICU	Mixed	51.56	31.88	71.25	0.64
	No	43.44	18.51	68.37	
Follow-up mode	Phone call	45.87	24.36	67.38	0.75
	Outpatient visits	40.81	13.71	67.90	
	Online survey	51.00	19.28	82.71	
	Mixed	62.87	32.03	93.70	
Sub-continents	East	5.06	1.99	12.31	< 0.0001
	Western	17.01	1.11	32.91	
	Southern	48.89	32.83	64.95	
	Northern	47.73	36.77	58.69	

## Discussion

To our knowledge, this is the first meta-analysis exploring prevalence, risk factors and symptomatology of long COVID in Africa. Twenty-five studies were included, above 1147 papers initially screened, for a total sample size of 29,213 patients. All the patients had a history of COVID-19 infection, confirmed by positive RT-PCR/NAAT (Nucleic Acid Amplification Test or NAAT) on nasopharyngeal swab associated with clinical manifestations and radiological findings.

Nearly 50% of the people included in this meta-analysis exhibited long COVID symptoms. This finding reinforces the critical significance of this emerging condition. In this study, fatigue was the most common symptom (35.4%, 95%CI 25.6–45.2) which represents the most debilitating long COVID symptom, and the first reason patients seek for medical assistance. This is concerning because, in Africa, it has the potential to lead to important impairment in productivity and further loss of economic agency.

In our study, females constituted 59.3% of the total population. However, we did not observe a significant association between gender and the incidence of any specific signs or symptoms of long COVID (Beta coefficient 0.04, *p* value interaction 0.41). These results contradict previous findings suggesting that females may be more susceptible to experiencing long COVID compared to males^18, 21^. Notably, significant research has indicated a higher occurrence of general, neurological, and cardiovascular symptoms, predominantly among females rather than males^19–23^.

In contrast, consistent with previous studies^24, 25, 27^, our findings support the notion that older age is a prominent factor associated with increased morbidity related to long COVID. Our analysis revealed a significant association between each additional year of age and a 10% higher probability of experiencing any signs or symptoms of long COVID, particularly in the areas of general health, psychiatric well-being, neurological function, and respiratory symptoms. These results indicate that, despite the relatively younger of the African population, advancing age continues to be a crucial risk factor for developing long COVID, even within this specific context.

Among people included in the analysis, prevalence of hospitalization and admission to ICU (Intensive Care Unit) was high, respectively 56.38 (95% CI 31.87–81.69) and 51.56 (95% CI 31.88–71.25). Meta-regression showed that percentage of hospitalization reported in each study significantly correlated with between a small increase in the prevalence of any long COVID symptomatology [Beta 0.003 (*p* = 0.048)]. This finding is in line with the meta-analysis conducted by Di Gennaro et al.^18^ over a population of 120,970 patients, and suggest that severity of the acute phase may play only a marginal role in the incidence of post-COVID conditions. In our study, the marginal role of acute phase severity was further underscored by the low R-squared value and by sensitivity analyses, that failed in demonstrating a correlation between incidence of long COVID and admission to ICU. However, potential confounders might be, among others, the profound differences between Africa and high-income countries—where most of the evidence about long COVID has been produced—in terms of both ICU access and availability of indicators used to define critical COVID-19, namely the need for high-flow nasal cannula, mechanical ventilation, ECMO or dialysis^26, 27^.

Furthermore, consistently with other studies^28, 29^, in the aftermaths of COVID-19 infection, up to a quarter of patients included in this study experienced Mental Health issues such as post-traumatic stress disorder (PTSD) or anxiety. This is concerning, because the additional burden in mental health disorder brought by the COVID-19 pandemic and its chronic consequences meets a health system which is largely unprepared to address mental health conditions. In Fact, a survey conducted by the WHO in 2014 revealed that only 55% of African countries had implemented independent mental health policies^30^. Furthermore, the region had a ratio of 1.4 mental health workers per 100,000 people, against a global average of 9.0 per 100,000, with a rate of patients visiting mental health facilities as low as 14 per 100,000—versus a mean of 1051 per 100,000 recorded for other regions^31^. These findings highlight the pressing need for immediate policy implementation and reallocation of resources to address this severely underestimated public health issue.

The results obtained about prevalence and key risk factors of long COVID occurrence might be useful and have serious implications for low-middle income countries of WHO African region, which have resource constrained health care systems. The evidence generated by this study will help the national public health response and strategy to reduce the impact of long COVID on quality of life, mental health and work ability. Many challenges have been enlightened in determining the prevalence of this condition in these settings, consequently the strategy might consist of improving the knowledge and the skills of health care workers in managing patients with any signs and symptoms of long COVID, updating clinical guidelines and implementing comprehensive healthcare services, particularly in major public healthcare facilities. Furthermore, it will be needed a widespread creation of supplementary community-based centers with qualified personnel where patients affected by this syndrome and with poor quality of life can acquire awareness about this condition and can be addressed to the rehabilitation process.

Several limitations should be acknowledged. First, although a close correlation with certain predisposing diseases or conditions has been established in several cohort studies and meta-analyses, we were not able to determine the impact of comorbidities and severe acute COVID-19 illness on the occurrence of long-term COVID syndrome. This was due to the high heterogeneity and fragmentation of the data collected in the included studies. Second, it is important to note that out of the 25 studies included in the analysis, only 7 were conducted in the WHO AFRO Region, while the remaining studies focused on North Africa. This disparity underscores the pressing need to generate high-quality evidence specifically within the Sub-Saharan African context. Third, it is crucial to acknowledge that the data regarding vaccination status and the specific COVID-19 variants were largely unknown, thereby hindering the ability to determine the influence of vaccination status on the incidence of long COVID across multiple waves.

Fourth, only English-language articles were considered in our meta-analysis and systematic review. Non-English publications, particularly Arabic publications, constitute a significant proportion of African medical literature, isolating African healthcare professionals from the most recent research. This language barrier also limits our knowledge and the reported data regarding long-term COVID symptoms in Africa.

## Conclusions

Long COVID is a major public health issue due to its prevalence in patients tested positive to SARS-COV-2 and the lack of effective therapeutic strategies. Low-middle-income countries do not generally have social safety nets, and the impact of chronic sequelae on the workforce and on families’ livelihoods remain a concern. In these countries, health care systems that need to also establish post-acute care services where physical, cognitive, and mental health disabilities will be recognized. More long-term, perspective studies are needed to understand the real long-term impact on quality of life and workforce activity and to develop optimal therapeutic and prevention strategies.

### Supplementary Information

Supplementary Information 1.

Supplementary Table 1.

## Data Availability

The study specific summary data included in the meta-analysis can be obtained from the corresponding authors at giacguido@gmail.com.
