# Supplementary material for: Incidence and burden of long COVID in Africa: a systematic review and meta-analysis
Source: Sci Rep. 2023 Dec 6;13:21482. doi: 10.1038/s41598-023-48258-3 (PMC10700349; doi:10.1038/s41598-023-48258-3)
Supplement: Supplementary file 1 — Supplementary Information 1. [file 41598_2023_48258_MOESM1_ESM.docx]

**Search entry:**

“(Africa OR Angola OR Algeria OR Benin OR Botswana OR Burkina Faso OR Burundi OR Cameroon OR Cape Verde OR Chad OR Central African Republic OR Comoros OR Ivory Coast OR Congo OR Egypt OR Eritrea OR Ethiopia OR Gabon OR Gambia OR Ghana OR Djibouti OR Guinea OR Kenya OR Lesotho OR Liberia OR Libya OR Madagascar OR Malawi OR Mali OR Mauritania OR Mauritius OR Morocco OR Mozambique OR Namibia OR Niger OR Nigeria OR Rwanda OR “São Tomé and Príncipe” OR Senegal OR Seychelles OR Sierra Leone OR Somalia OR South Africa OR Sudan OR eSwatini OR Tanzania OR Togo OR Tunisia OR Uganda OR Zambia OR Zimbabwe) AND ("COVID-19" OR "Novel Coronavirus–Infected Pneumonia" OR "2019 novel coronavirus" OR "2019-nCoV" OR "SARS-CoV-2") AND ("lingering symptoms" OR "persistent symptoms" OR "long-term symptoms" OR "long-term Covid" OR "long-term" OR "long term" OR "long")
