# Supplementary material for: Incidence and burden of long COVID in Africa: a systematic review and meta-analysis
Source: Sci Rep. 2023 Dec 6;13:21482. doi: 10.1038/s41598-023-48258-3 (PMC10700349; doi:10.1038/s41598-023-48258-3)
Supplement: Supplementary file 2 — Supplementary Table 1. [file 41598_2023_48258_MOESM2_ESM.docx]

**Supplementary Table 1. Descriptive characteristics**

| **Author** | **Year** | **Country** | **Sample size** | **Mean age** | **Number of people hospitalized** | **Number of people admitted to ICU** | **Methods for COVID19 diagnosis** | **Follow-up mode** | **Follow-up (months)** | **Number of people vaccinated** | **COVID main variant** | **NOS** |
| --- | --- | --- | --- | --- | --- | --- | --- | --- | --- | --- | --- | --- |
| Abate Yeshidinber Weldetsadik^32^ | 2022 | Ethiopia | 79 | 6.9 | 79 | 6 | PCR | Phone call | 3 | Unknown | Unknown | 5 |
| Abdelrahman^33^ | 2021 | Egypt | 172 | 41.8 | 22 | 7 | PCR | Phone call | 12 | Unknown | Unknown | 6 |
| Ahmed^34^ | 2021 | Egypt | 300 | 46.49 | 300 | Unknown | PCR | Outpatient visit / in person interview | 6 | Unknown | Unknown | 6 |
| Aly^35^ | 2021 | Egypt | 115 | 73.18 | 115 | 0 | PCR | Online survey | 1 | Unknown | Unknown | 6 |
| Charfeddine^36^ | 2021 | Tunisia | 798 | 49.94 | 798 | 0 | PCR | Mixed | 6 | Unknown | Unknown | 6 |
| Crankson, S. and Pokhrel, S. and Anokye, N. K.^37^ | 2022 | Ghana | 2334 | 40.2 | Unknown | 0 | PCR | Outpatient visit / in person interview | 1 | Unknown | Unknown | 6 |
| Dryden^10^ | 2022 | South Africa | 1873 | 52 | 1873 | 612 | PCR | Phone call | 3 | Unknown | Beta | 6 |
| El Otmani^38^ | 2022 | Morocco | 236 | 29 | Unknown | 0 | Mixed (clinical and microbiological) | Online survey | 3 | 121 | Unknown | 5 |
| Elanwar^39^ | 2021 | Egypt | 92 | 52.8 | Unknown | 0 | PCR | Outpatient visit / in person interview | 6 | Unknown | Unknown | 6 |
| Elmazny^40^ | 2023 | Egypt | 1638 | 38.28 | Unknown | 0 | PCR | Phone call | 3 | 305 | Unknown | 6 |
| Hend Ibrahim Shousha^41^ | 2021 | Egypt | 199 | Unknown | 172 | 0 | PCR | Online survey | 6 | Unknown | Unknown | 6 |
| James Exnobert Zulu^42^ | 2022 | Zambia | 302 | 32 | 0 | 0 | PCR | Outpatient visit / in person interview | 2 | Unknown | Unknown | 5 |
| Magdy DM^43^ | 2022 | Egypt | 85 | 34.6 | 85 | 25 | PCR | Outpatient visit / in person interview | 6 | Unknown | Unknown | 7 |
| Magdy Rehab^44^ | 2022 | Egypt | 408 | 33 | Unknown | Unknown | PCR | Mixed | 3 | 75 | Unknown | 6 |
| Marwa Kamal^45^ | 2021 | Egypt | 287 | 32.3 | 287 | 14 | NR | Online survey | 5 | Unknown | Unknown | 6 |
| Marwa Khalaf^46^ | 2022 | Egypt | 538 | 41.17 | 276 | 35 | NR | Mixed | 8 | 0 | Unknown | 5 |
| Mendelsohn AS^47^ | 2022 | South Africa | 174 | 50.3 | 3 | 0 | PCR | Phone call | 2 | Unknown | Beta | 6 |
| Mohamed-Hussein AAR^48^ | 2021 | Egypt | 262 | Unknown | 95 | Unknown | NR | Online survey | 3 | Unknown | Unknown | 6 |
| Nassim Essabah Haraj^49^ | 2020 | Morocco | 41 | 55 | 41 | 31 | PCR | Online survey | 1 | Unknown | Unknown | 6 |
| O Evbuomwan^50^ | 2022 | South Africa | 78 | Unknown | 0 | 0 | NR | Outpatient visit / in person interview | 12 | Unknown | Beta | 7 |
| Osikomaiya B.^51^ | 2021 | Nigeria | 274 | Unknown | 274 | Unknown | PCR | Outpatient visit / in person interview | 0.5 | Unknown | Unknown | 5 |
| Schaalan Mona^52^ | 2022 | Egypt | 15166 | Unknown | 857 | Unknown | PCR | Online survey | 4 | Unknown | Unknown | 6 |
| Waasila Jassat^6^ | 2023 | South Africa | 2626 | 49 | Unknown | 787 | Mixed (clinical and microbiological) | Outpatient visit / in person interview | 6 | 1914 | Beta | 7 |
| Waasila Jassat^6^ | 2023 | South Africa | 1074 | 37 | Unknown | 0 | Mixed (clinical and microbiological) | Outpatient visit / in person interview | 6 | 623 | Delta | 7 |
| Wose Kinge C^53^ | 2022 | South Africa | 62 | Unknown | 7 | 3 | NR | Online survey | 3 | Unknown | Beta | 6 |

REFERENCES

32. Weldetsadik AY, Abayneh M, Abraha M, Betizazu SS, Bekele D. Clinical Characteristics and Outcome of Pediatric COVID-19 Patients in Ethiopia During the Early COVID-19 Pandemic: A Prospective Cohort Study. *Pediatr Health Med Ther*. 2022;Volume 13:165-174. doi:10.2147/PHMT.S359333

33. Abdelrahman MM, Abd‐Elrahman NM, Bakheet TM. Persistence of symptoms after improvement of acute COVID19 infection, a longitudinal study. *J Med Virol*. 2021;93(10):5942-5946. doi:10.1002/jmv.27156

34. Ahmed GK, Khedr EM, Hamad DA, Meshref TS, Hashem MM, Aly MM. Long term impact of Covid-19 infection on sleep and mental health: A cross-sectional study. *Psychiatry Res*. 2021;305:114243. doi:10.1016/j.psychres.2021.114243

35. Aly MAEG, Saber HG. Long COVID and chronic fatigue syndrome: A survey of elderly female survivors in Egypt. *Int J Clin Pract*. 2021;75(12). doi:10.1111/ijcp.14886

36. Charfeddine S, Ibn Hadj Amor H, Jdidi J, et al. Long COVID 19 Syndrome: Is It Related to Microcirculation and Endothelial Dysfunction? Insights From TUN-EndCOV Study. *Front Cardiovasc Med*. 2021;8:745758. doi:10.3389/fcvm.2021.745758

37. Crankson S, Pokhrel S, Anokye NK. Determinants of COVID-19-Related Length of Hospital Stays and Long COVID in Ghana: A Cross-Sectional Analysis. *Int J Environ Res Public Health*. 2022;19(1):527. doi:10.3390/ijerph19010527

38. El Otmani H, Nabili S, Berrada M, Bellakhdar S, El Moutawakil B, Abdoh Rafai M. Prevalence, characteristics and risk factors in a Moroccan cohort of Long-Covid-19. *Neurol Sci*. 2022;43(9):5175-5180. doi:10.1007/s10072-022-06138-0

39. Elanwar R, Hussein M, Magdy R, et al. Physical and Mental Fatigue in Subjects Recovered from COVID-19 Infection: A Case–Control Study. *Neuropsychiatr Dis Treat*. 2021;Volume 17:2063-2071. doi:10.2147/NDT.S317027

40. Elmazny A, Magdy R, Hussein M, et al. Neuropsychiatric post-acute sequelae of COVID-19: prevalence, severity, and impact of vaccination. Eur Arch Psychiatry Clin Neurosci. 2023;273(6):1349-1358. doi:10.1007/s00406-023-01557-2

41. Shousha HI, Madbouly N, Afify S, et al. Anxiety, depression and coping strategies among chronic medical patients with coronavirus disease-2019: a multicenter follow-up cohort study. *J Ment Health*. Published online September 30, 2021:1-9. doi:10.1080/09638237.2021.1979491

42. Zulu JE, Banda D, Hines JZ, et al. Two-month follow-up of persons with SARS-CoV-2 Infection-Zambia, September 2020: a cohort study. *Pan Afr Med J*. 2022;41. doi:10.11604/pamj.2022.41.26.30721

43. Magdy D, Metwally A, Tawab D, Hassan S, Makboul M, Farghaly S. Long-term COVID-19 effects on pulmonary function, exercise capacity, and health status. *Ann Thorac Med*. 2022;17(1):28. doi:10.4103/atm.atm_82_21

44. Magdy R, Elmazny A, Soliman SH, et al. Post-COVID-19 neuropsychiatric manifestations among COVID-19 survivors suffering from migraine: a case–control study. *J Headache Pain*. 2022;23(1):101. doi:10.1186/s10194-022-01468-y

45. Kamal M, Abo Omirah M, Hussein A, Saeed H. Assessment and characterisation of post‐COVID‐19 manifestations. *Int J Clin Pract*. 2021;75(3). doi:10.1111/ijcp.13746

46. Khalaf M, Alboraie M, Abdel-Gawad M, et al. Prevalence and Predictors of Persistent Symptoms After Clearance of SARS-CoV-2 Infection: A Multicenter Study from Egypt. *Infect Drug Resist.* 2022;15:2575-2587. Published 2022 May 20. doi:10.2147/IDR.S355064

47. Mendelsohn, A. S., Nath, N., De Sá, A., & Von Pressentin, K. B. (2022). Two months follow-up of patients with non-critical COVID-19 in Cape Town, South Africa. [*S Afr Fam Pract*](https://www.ncbi.nlm.nih.gov/pmc/articles/PMC8905322/), *64*(1), e1–e6. Published 2022 Feb 10. doi:10.4102/safp.v64i1.5429

48. Mohamed-Hussein AAR, Amin MT, Makhlouf HA, et al. Non-hospitalised COVID-19 patients have more frequent long COVID-19 symptoms. *Int J Tuberc Lung Dis*. 2021;25(9):732-737. doi:10.5588/ijtld.21.0135

49. Haraj NE, El Aziz S, Chadli A, et al. Nutritional status assessment in patients with Covid-19 after discharge from the intensive care unit. *Clin Nutr ESPEN*. 2021;41:423-428. doi:10.1016/j.clnesp.2020.09.214

50. Evbuomwan O, Endres W, Tebieia T, Engelbrecht G. Incidence and follow-up of persistent lung perfusion abnormalities as a result of suspected air trapping or microthrombosis in non-hospitalised COVID-19 patients during the early half of the pandemic – experience in a tertiary institution in South Afr. *S Afr Med J*. Published online November 1, 2022:850-854. doi:10.7196/SAMJ.2022.v112i11.16578

51. Osikomaiya B, Erinoso O, Wright KO, et al. ‘Long COVID’: persistent COVID-19 symptoms in survivors managed in Lagos State, Nigeria. *BMC Infect Dis*. 2021;21(1):304. doi:10.1186/s12879-020-05716-x

52. Schaalan M, Abou Warda AE, Osman SM, et al. The Impact of Sociodemographic, Nutritional, and Health Factors on the Incidence and Complications of COVID-19 in Egypt: A Cross-Sectional Study. *Viruses*. 2022;14(3):448. doi:10.3390/v14030448

53. Wose Kinge C, Hanekom S, Lupton-Smith A, et al. Persistent Symptoms among Frontline Health Workers Post-Acute COVID-19 Infection. *Int J Environ Res Public Health*. 2022;19(10):5933. doi:10.3390/ijerph19105933
